# The Different Effects of Direct Bilirubin on Portopulmonary Hypertension and Idiopathic Pulmonary Arterial Hypertension

**DOI:** 10.1155/2022/7021178

**Published:** 2022-02-03

**Authors:** Yuan Li, Hongling Qiu, Qinhua Zhao, Jing He, Rong Jiang, Wenhui Wu, Cijun Luo, Huiting Li, Lan Wang, Jinming Liu, Sugang Gong

**Affiliations:** Department of Pulmonary Circulation, Shanghai Pulmonary Hospital, School of Medicine, Tongji University, Shanghai, China

## Abstract

**Background:**

To observe different roles of direct bilirubin (Dbil) on portopulmonary hypertension (POPH) and idiopathic pulmonary arterial hypertension (IPAH).

**Methods:**

Thirty incident patients with POPH and 180 with IPAH (matched by the WHO functional classification in a 1 : 6 ratio) between March 2010 and December 2020 were included. The receiver operating curve and Kaplan–Meier method were applied to estimate the ability to distinguish between the two and survival, respectively. Univariate and forward multiple stepwise regression analyses were performed to access the relationship between pulmonary vascular resistance (PVR) and clinical indices.

**Results:**

Compared to IPAH, the POPH group had better hemodynamics including PVR (7.08 ± 3.95 vs. 14.89 ± 7.11, *P* < 0.001) and higher total bilirubin (Tbil) and Dbil. Tbil and Dbil had a negative correlation with PVR in the POPH group (*r* = −0.394, *P*=0.031; *r* = −0.364, *P*=0.048, respectively) but positive correlation in the IPAH group (*r* = 0.218, *P*=0.003; *r* = 0.178, *P*=0.018, respectively). Increased neutrophil counts (*r* = 0.394, *P*=0.031) and elevated NT-proBNP (*r* = 0.433, *P* < 0.001) would help predict the elevation of PVR in POPH and IPAH groups independent of Dbil, respectively. Dbil could distinguish POPH from IPAH (AUC = 0.799, *P*=0.009), and the ability was elevated when taking aspartate aminotransferase together (AUC = 0.835, *P* < 0.001). The overall survival was better in POPH than in IPAH (7 dead cases of POPH and 96 of IPAH, *P*=0.002). Survival was better in POPH than in IPAH in the group of Dbil ≥7 *μ*mol/L (*P*=0.001) but showed no significant difference between POPH and IPAH in the group of Dbil <7 *μ*mol/L (*P*=0.192).

**Conclusions:**

The POPH group had a better hemodynamic profile than IPAH. Dbil was associated oppositely with the elevation of PVR in POPH and IPAH. Patients with POPH had better survival than those with IPAH in the total cohort and in the group of Dbil ≥7 *μ*mol/L, but limited dead cases of POPH should be noted.

## 1. Introduction

Portopulmonary hypertension (POPH) is a life-threatening disease with damage to both pulmonary circulation and portal circulation with or without liver diseases, defined as Group 1 pulmonary hypertension (PH) and a severe complication of portal hypertension [1–5]. According to epidemiology, POPH could explain 5–15% of pulmonary arterial hypertension (PAH) causing a specific associated PAH form [6, 7]. Hemodynamic studies showed that 2–6% of patients with portal hypertension had significantly obvious pulmonary hypertension [8]. To date, pieces of literature regarding POPH stay a few, but POPH has still not been well recognized. In spite of many puzzles of POPH, POPH was generally thought to share some similar pathobiological mechanisms to other forms of PAH [2, 9].

Bilirubin, including total bilirubin and direct bilirubin, is one indicator of liver function abnormality and had an association with PAH. During the 1990s, higher expression levels of total bilirubin were found to be a risk factor for early postoperative mortality in 31 patients with primary PH and 31 patients with Eisenmenger syndrome who all underwent heart-lung transplantation [10]. Meanwhile, Takeda et al. [11] and colleagues conducted the research regarding bilirubin and mortality in 18 patients with idiopathic PAH (IPAH) and 19 with connective tissue disease-associated PAH (CTD-PAH) and found hyperbilirubinemia and total bilirubin concentration to be risk predictors of death independently of WHO functional classification and brain natriuretic peptide (BNP), respectively. Our previous study investigated 404 patients with IPAH at enrollment. The results suggested that the expression level of direct serum bilirubin was much higher in nonsurvivors than in survivors, and the baseline expression level of direct serum bilirubin could predict severity and outcomes of IPAH [12].

POPH, as a disease related to liver abnormality, probably exists in abnormal bilirubin. However, the comparisons of bilirubin between POPH and IPAH stay unclear. Thus, our objective was to make comparisons between IPAH and POPH.

## 2. Materials and Methods

### 2.1. Ethics and Population

This study was approved by the Ethics Committee of the Shanghai Pulmonary Hospital with the approval number k16-293 and according to the Declaration of Helsinki. Written consent was obtained from each patient.

**Figure 1:**
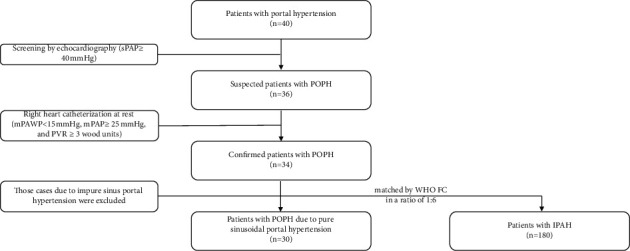
The flowchart of POPH confirmation, the inclusion and the exclusion. POPH, portopulmonary hypertension; IPAH, idiopathic pulmonary arterial hypertension. sPAP, systolic pulmonary arterial pressure; mPAP, mean pulmonary arterial pressure; mPAWP, mean pulmonary artery wedge pressure; PVR, pulmonary vascular resistance; WHO FC, World Health Organization functional classification.

Patients with portal hypertension were diagnosed at other centers and would come to our center for PH confirmation. The acute reports regarding hepatic venous portal pressure gradient (HVPG) values were not obtained. All patients were firstly screened by echocardiography (systolic pulmonary artery pressure ≥40 mmHg) and then underwent right heart catheterization (RHC) to confirm PAH at our center. There were 34 cases with POPH confirmed at our center from March 2010 to December 2020. Of those, 2 cases with Budd-Chiari syndrome and 2 cases with schistosomiasis were mechanically different from the other 30 cases (pure sinusoidal portal hypertension) and were excluded from the study. To include a total of 30 patients with POPH in this retrospective study, patients with IPAH were matched by WHO functional classification (WHO FC) in a 1 : 6 ratio to generate a typical landscape of IPAH and increase the test power. The flow diagram is shown in Figure 1. The diagnosis of IPAH and POPH was made in accordance with the standard guideline as to the following. The criteria for IPAH included (i) mean pulmonary arterial pressure (mPAP) ≥25 mmHg, mean pulmonary arterial wedge pressure (mPAWP) ≤15 mmHg, and pulmonary vascular resistance (PVR) ≥3 wood units measured at rest through RHC; (ii) exclusion of pulmonary hypertension due to left heart disease or lung diseases or chronic thromboembolism pulmonary hypertension; and (iii) exclusion of other forms of PAH via special tests containing hematology, biochemistry, immunology, serology, ultrasound, etc. The diagnosis of POPH was according to (i) mPAP ≥ 25 mmHg, mPAWP < 15 mmHg, and PVR ≥ 3 wood units measured at rest through RHC and (ii) portal hypertension (ascites, splenomegaly, and varicose veins).

### 2.2. Demographics, Hemodynamics, Clinical Variables, and Outcome Collection

Demographics, World Health Organization functional classification (WHO FC), target therapy, liver function, etiological originals of portal hypertension, and other related data were collected. The MELD (model of end-stage liver disease) equation was applied to calculate the score for severity as 9.57 × ln (creatinine, mg/dL) + 3.78 × ln (total bilirubin, mg/dL) + 11.20 × ln (international normalized ratio) + 6.43. The minimal values were forced to 1.0 for calculation purposes [13]. Baseline hemodynamics were measured via RHC at rest in all patients. The patients were followed up until 23 December 2020 through telephone interviews and outpatient clinic visits. All-cause mortality was the observational endpoint. During the period of follow-up, no one was lost in this study.

### 2.3. Data Analysis and Statistics

Continuous variables with normal distribution were expressed as mean ± SD, and the independent Student's *t*-test was used for the comparisons. Continuous variables with nonnormal distribution were expressed as median (first and third interquartile), and the Mann-Whitney *U* test was applied for the comparisons. Categorical variables were expressed as the number of patients and relative frequencies (*n*, %), and the Chi-square test was used for the comparisons. The receiver operating characteristic (ROC) curve was employed to assess the ability of distinguishing POPH from IPAH. The predictive ability in the models should be compared with integrated discrimination improvement (IDI) [14] and net reclassification improvement (NRI) [15]. Correlations were generated by Pearson or Spearman correlation analyses. Univariate and forward multiple stepwise regression analysis was performed to assess the relationship of PVR and clinical indices, which were adjusted by gases of blood, metabolic comorbidities, Child-Pugh score, and concomitant medications.

Overall survival time was defined from the date of diagnostic RHC to death. Surviving patients were censored on the date of the last clinical contact. The Kaplan–Meier method was used to estimate the proportion of patients surviving at each time point. Survival curves were compared with the log-rank test. All comparisons were employed with a two-sided test through SPSS (Statistical Package for Social Science, Chicago, IL) version 22.0, and a *P* value less than 0.05 was considered significant. All figures were concluded via GraphPad Prism (San Diego, CA, USA) version 7.0 software.

## 3. Results

### 3.1. Demographic and Clinical Indices at Baseline

A total of 30 POPH (8 men) and 180 IPAH (57 men) patients were included in this study. The mean time of follow-up was 70.23 ± 41.74 months. Data are illustrated in Table 1. Patients with POPH were older than patients with IPAH (49.5 ± 13.1 vs. 39.1 ± 14.9 years old, *P* < 0.001). In patients with POPH, the most common etiology of liver disease was hepatitis B virus infection (19 cases, 63.4%) and the second one was cryptogenic disease (7 cases, 23.3%). The Child-Pugh class of POPH was mainly distributed in class A (13 cases, 43.3%) and class B (14 cases, 46.7%). D-dimer (399 (218, 1673) vs. 149 (106, 202) ng/ml, *P* < 0.001) and PCO_2_ (33.96 ± 11.68 vs. 28.43 ± 5.07 mmHg, *P*=0.019) were significantly higher, while NT-proBNP (324 (81, 893) vs. 739 (262, 1904) pg/ml, *P*=0.004) was significantly lower in the POPH group rather than in IPAH group. Compared to the IPAH group, patients in the POPH group were more likely to receive the treatment of phosphodiesterase 5 inhibitors (19 cases (63.2%) vs. 77 cases (42.7%), *P*=0.036).

In the aspect of arterial blood gas, patients with POPH had higher PCO_2_ (33.96 ± 11.68 vs. 28.43 ± 5.07 mmHg, *P*=0.019) than patients with IPAH. With respect to blood cell counts, the POPH group had lower white blood cell counts (4.18 ± 1.78 vs. 6.68 ± 2.09 10^9^/L, *P* < 0.001), neutrophil counts (2.26 ± 1.40 vs. 3.88 ± 1.79 10^9^/L, *P* < 0.001), red blood cell counts (2.26 ± 1.40 vs. 3.88 ± 1.79 10^9^/L, *P* < 0.001), and platelet counts (114.73 ± 85.67 vs. 178.92 ± 72.54 10^9^/L, *P* < 0.001) than the IPAH group.

Hemodynamic variables were quite different between POPH and IPAH. Compared to patients with IPAH, patients with POPH had lower systolic pulmonary artery pressure (sPAP, 80.46 ± 21.71 vs. 99.25 ± 24.81 mmHg, *P* < 0.001), mean right atrial pressure (mRAP, 4.74 ± 3.54 vs. 8.48 ± 5.49 mmHg, *P* < 0.001), mean pulmonary artery pressure (mPAP, 46.54 ± 11.95 vs. 60.58 ± 15.03 mmHg, *P* < 0.001), pulmonary vascular resistance (PVR, 7.01 ± 4.07 vs. 14.82 ± 7.00 wood unit, *P* < 0.001), transpulmonary gradient (TPG, 38.24 ± 11.21 vs. 52.38 ± 14.77 mmHg, *P* < 0.001), and systemic vascular resistance (SVR, 15.78 ± 7.37 vs. 22.01 ± 8.23 wood unit, *P*=0.001) but higher cardiac index (CI, 4.02 ± 1.59 vs. 2.47 ± 0.81 L/min/m^2^, *P* < 0.001) and mixed venous oxygen saturation (SvO_2_, 65.50 ± 9.73 vs. 60.97 ± 10.00%, *P*=0.004). Of these differential variables, the gap of PVR between the POPH group and the IPAH group exhibited a two-fold change.

### 3.2. Variables of the Liver Function Test

Patients with POPH showed significantly higher expression of total bilirubin (25.0 (17.0, 44.3) vs. 19.0 (14.0, 28.3) *μ*mol/L, *P*=0.013), direct bilirubin (10.5 (6.0, 17.0) vs. 6.0 (4.0, 10.5) *μ*mol/L, *P*=0.013), and aspartate aminotransferase (AST, 37.5 (30.0, 44.0) vs. 25.0 (20.0, 30.0) IU/L, *P* < 0.001) and lower expression of albumin (32.88 ± 4.27 vs. 38.41 ± 5.14 g/L, *P* < 0.001) than patients with IPAH. In addition, alanine aminotransferase (ALT, 26.5 (17.8, 38.0) vs. 24.0 (19.0, 32.0) IU/L, *P*=0.566) showed no difference between the POPH group and IPAH group. These data are generated in Table 2.

### 3.3. Correlation between PVR and Differential Laboratory Variables and Liver Function Test in POPH and IPAH

Table 3 illustrates the correlations between PVR and the clinical variables and indices in POPH and IPAH. (PVR, total bilirubin, direct bilirubin, AST, D-dimer, blood cell counts, NT-proBNP, and PCO_2_ were regarded as continuous variables. Target therapies and MELD scores were deemed as categorical variables.) The analysis found that total bilirubin and direct bilirubin presented a negative correlation with PVR in the POPH group (*r* = −0.394, *P*=0.031; *r* = −0.364, *P*=0.048, respectively), while they showed a positive correlation with PVR in the IPAH group (*r* = 0.218, *P*=0.003; *r* = 0.178, *P*=0.018, respectively). NT-proBNP was positively related to PVR in the IPAH group (*r* = 0.438, *P* < 0.001), and neutrophil counts had a positive correlation with PVR in the POPH group (*r* = 0.394, *P*=0.031).

### 3.4. Independent Determinants to Predict PVR Elevation in POPH and IPAH

Taking those potentially correlated variables (the significance is shown in Table 3) into account, we applied a multiple stepwise regression analysis to determine the strength of the prediction of the elevation of the PVR (as a continuous variable) after adjusting for gases of blood (continuous variable), metabolic comorbidities (yes or not, categorical variable, including hypertension, diabetes, obesity, and dyslipidaemia), Child-Pugh score (categorical variable), and concomitant medications (categorical variable). In the POPH group, elevated neutrophil counts as an independent predictor purported rising PVR accounting for 15.6% change (standardized *β* = 0.394, *P*=0.030). NT-proBNP had the sole role in positively predicting PVR elevation in the IPAH group and made 18.7% variation clear (standardized *β* = 0.433, *P* < 0.001). This information is illustrated in Table 4.

### 3.5. Ability of Direct Bilirubin to Identify POPH from Total Patients

**Figure 2:**
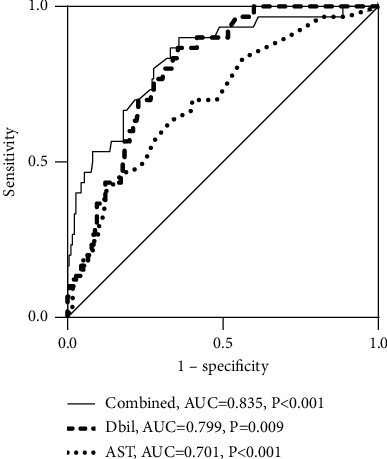
The ability of direct bilirubin (Dbil), aspartate aminotransferase (AST), and their combination to distinguish POPH from the total cohort. POPH, portopulmonary hypertension; IPAH, idiopathic pulmonary arterial hypertension; AUC, area under the curve.

ROC analysis was performed to assess the ability of direct bilirubin, AST, and their combination to identify POPH from total patients (Figure 2). We found the significant strength of direct bilirubin in distinguishing POPH from total patients with AUC = 0.799 and *P*=0.009. Meanwhile, another abnormal liver function index of AST also showed the significant ability to identify POPH from IPAH, of which the AUC was 0.701 (*P* < 0.001). When combining the two variables together, the ability to identify POPH from IPAH had been improved and the AUC was elevated to 0.835 (*P* < 0.001). We also calculated the value of IDI and NRI to evaluate the elevated ability of the combined model relative to the direct bilirubin model, and the results were following: absolute IDI = 0.064, relative IDI = 0.074, and NRI = 0.030.

### 3.6. Survival Assessment between POPH and IPAH

**Figure 3:**
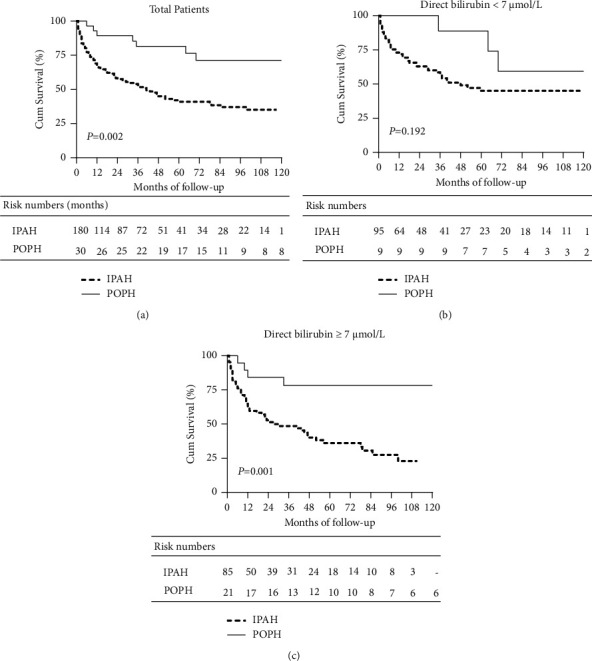
Comparison of estimated survival between IPAH and POPH in total patients (a). When divided by the upper limit of the normal range of direct bilirubin (both IPAH and POPH), the different survival situations between POPH and IPAH patients with direct bilirubin <7 *μ*mol/L (b) and patients with direct bilirubin ≥7 *μ*mol/L (c). POPH, portopulmonary hypertension; IPAH, idiopathic pulmonary arterial hypertension.

There were a total of 103 dead cases (7 POPH) during the follow-up. Of those dead cases, 72 patients with IPAH (75.0%) and 2 patients with POPH (28.6%) died of right heart failure. With respect to liver disease, no one died of liver failure in IPAH, while 5 patients with POPH (71.4%) died of liver failure. The 1st-year, 3rd-year, 5th-year, and 10th-year overall survival rates were found to be 67.2%, 53.2%, 41.0%, and 35.2% in the IPAH group and 89.3%, 81.5%, 81.5%, and 71.3% in the POPH group, respectively (log-rank = 0.002, Figure 3(a)). With the division of direct bilirubin (both IPAH and POPH), we found that there was no significantly different survival between POPH and IPAH in the group of direct bilirubin <7 *μ*mol/L (log-rank = 0.192, Figure 3(b)), but it showed better survival in POPH than in IPAH in the group of direct bilirubin ≥7 *μ*mol/L (log-rank = 0.001, Figure 3(c)).

## 4. Discussion

To date, there have still been limited types of literature talking about the differences in bilirubin between IPAH and POPH. Our study demonstrated some interesting findings as follows: (I) when matched by WHO FC, patients with POPH had better hemodynamics and survival than patients with IPAH; (II) patients with POPH had worse liver function than patients with IPAH, which showed that the medians (first and third interquartile) of direct bilirubin were 10.5 (6.0, 17.0) and 6.0 (4.0, 10.5) *μ*mol/L, respectively; (III) total bilirubin and direct bilirubin associated with PVR positively in the IPAH group but negatively in the POPH group; (IV) elevated neutrophil counts and elevated NT-proBNP were independent predictors of PVR increase in POPH and IPAH, respectively; (V) direct bilirubin with AST could better help identify POPH from IPAH than separate direct bilirubin or AST.

No matter from the REHAP registry [16], the REVEAL registry [17], or the data of the National Research Project on Intractable Disease in Japan [18], POPH subjects were considered with better hemodynamics, which was similar to our data. However, the western population [13, 14] showed a worse prognosis in POPH than in IPAH or heritable PAH (HPAH) when referring to the survival situation. In contrast, patients with POPH from the Japanese population showed no significant difference in survival compared to IPAH ones [18]. Our study showed better survival in POPH rather than in IPAH when the populations were limited into a similar WHO functional classification. Although a recent study from China [19] had shown a trend similar to the western population, the limited cases of 10 PHT-post-splenectomy-PH and 20 IPAH were not that easy to generate the typical landscape of IPAH. Our study contained 30 cases with POPH and 180 cases with IPAH, which could help recognize the difference between POPH and a typical landscape of IPAH. It is easy to consider that race might be an indicator of different survival among countries and regions. However, a study from the REVEAL registry, including a total of 3046 patients in which 100 were deemed as Asian or Pacific Islander, demonstrated that the race was not significantly associated with survival after age under 60 years adjustment [20]. Meanwhile, DuBrock's study [21] found that patients with POPH had lower socioeconomic status than patients with IPAH, in which lower education level would associate with more emergency department visits in American patients. Different survival among countries and regions requires more clinical investigations concerning the development of economy and medication, diets, mental situation, the spectrum of primary diseases, etc. Another difference in our study from the western population was the etiology of liver disease. Our data showed that the most etiology was hepatitis B virus infection of POPH rather than alcohol. Actually, the burden of liver diseases differed a lot from countries and regions [22]. In the western population, alcohol was the major cause of liver diseases, including POPH, while hepatitis B virus infection was higher than alcohol factor in East Asia [22, 23]. Meanwhile, our study was with a small sample size, which means that the small dead cases of POPH limited the survival comparison between POPH and IPAH. A new national study is expected in the future.

We chose PVR to conduct the correlation analysis not only because of the two-time fold change between POPH and IPAH but also because PVR could predict mortality and graft failure in transplantation patients with POPH [24]. Although total bilirubin and direct bilirubin were positively correlated with PVR, the independent predictor of PVR elevation was NT-proBNP in the IPAH group. Bilirubin has been considered to positively correlate with the hemodynamic profile in patients with heart failure (HF) [25]. And then, total bilirubin was among the most highly significant predictors of mortality in a large cohort of chronic HF patients in a clinical trial [26]. As aware, right HF was the leading cause of death of IPAH [27, 28], and NT-proBNP is one of the most valuable biomarkers for diagnosing HF. Furthermore, a prior study has demonstrated a positive correlation between serum bilirubin concentration and lognormal concentration of BNP [11]. Therefore, it is not difficult to understand the positive correlation between bilirubin and the independent predicted value of NT-proBNP to the elevation of PVR in the IPAH group. However, there was no significant association between NT-proBNP and PVR in the POPH group, and it indicated that HF might not be the main cause of death in POPH patients when the most likely cause of death in POPH patients was liver disease. MELD scores could present the severity of liver disease, and it was deemed as a predictor of mortality in POPH [29]. However, we found that MELD scores had no significant correlation with PVR in POPH when the previous study found that MELD scores correlated poorly with PVR (*r* = −0.01) [30]. The differences might come from the small sample size in our study and the measured means of echocardiography in the later study.

The negative correlation between PVR and direct bilirubin was a decent finding in the POPH group. Horsfall [31] and colleagues conducted an extensive, statin-treated cohort (without liver disease or cardiovascular disease) research and found that low expression levels of serum bilirubin were associated with an increased risk of cardiovascular disease, suggesting a beneficial effect of elevated bilirubin levels. Meanwhile, a cohort containing 504,206 adults from a UK primary care research database showed the negative association between serum bilirubin and incidence of respiratory disease (chronic obstructive pulmonary disease and lung cancer) and all-cause mortality [32]. In the general population of Korea, the association between serum bilirubin and cardiovascular disease exhibited a trend similar to the before two [33]. In fact, bilirubin was thought to have antioxidant and anti-inflammatory effects over the past decades [34, 35], in which a bilirubin-biliverdin cycling mechanism could help explain the biological effects [36]. In in vitro experiments, Mazzone et al. [37] found that Human Umbilical Vein Endothelial Cells (HUVECs) prevented the adhesion induced by TNF*α* after being treated with different serum unconjugated bilirubin concentrations, in which the expression of E-selectin VCAM-1 and ICAM-1 was reduced.

Meanwhile, the neutrophil counts were the independent predictor of PVR elevation in POPH after taking total bilirubin, direct bilirubin, and neutrophil counts into the linear regression analysis. In fact, bilirubin had some association with neutrophils in some pulmonary diseases. Biliverdin, which can be interconverted with bilirubin, improved pulmonary inflammation induced by hemorrhagic shock and resuscitation (HSR). The authors used biliverdin treatment before HSR in rats and found markedly decreased neutrophil infiltration in the lung sections (neutrophils were stained by the naphthol AS-D chloroacetate method) compared to the sham group [38]. In the mouse model of chronic obstructive pulmonary disease induced from cigarette smoke exposure (CSE), the liver growth factor (LGF), an albumin-bilirubin complex, exhibited the antifibrotic, antioxidant, and antihypertensive actions at extrahepatic sites. After CSE mice were treated with LGF, the circulating T lymphocytes were significantly decreased and neutrophils from peripheral blood tended to be reduced compared to the CSE group [39]. Nevertheless, whether the bilirubin would influence the pathological and physiological changes of POPH and IPAH is still unknown, and more studies exploring the different mechanisms between POPH and IPAH should be conducted.

## 5. Conclusions

When matched by WHO FC, POPH patients had a distinct demographic, clinical, and hemodynamic profile compared to IPAH patients. Total bilirubin and direct bilirubin had a negative correlation with PVR in POPH but positive in IPAH. Patients with POPH had better survival than IPAH patients in the group of total patients and the group of patients with direct bilirubin ≥7 *μ*mol/L, but the limited dead cases of POPH should be noted.

## Figures and Tables

**Table 1:** Baseline demographics and clinical indices of portopulmonary hypertension and idiopathic pulmonary arterial hypertension.

Parameters	POPH (*n* = 30)	IPAH (*n* = 180)	*P* value
Demographics			
Age at diagnosis (year)	49.5 ± 13.1	39.1 ± 14.9	<0.001
Female : male	22 : 8	123 : 57	0.854
Height (cm)	162.7 ± 7.6	161.9 ± 6.9	0.584
Weight (kg)	63.0 ± 12.9	58.5 ± 9.9	0.032
Body surface area (m^2^)	1.70 ± 0.29	1.62 ± 0.18	0.046
Onset of symptoms (mo)	23 (10–37)	19 (9–44)	0.856
WHO FC (I : II : III : IV)	1 : 12 : 14 : 3	2 : 61 : 107 : 10	0.571
Etiology of liver disease, *n* (%)			
Cryptogenic	7 (23.3%)	NA	
Hepatitis B	19 (63.4%)	NA	
Hepatitis C	1 (3.3%)	NA	
Alcohol	1 (3.3%)	NA	
PBC	2 (6.7%)	NA	
Child-Pugh class, *n* (%)			
A	13 (43.3%)	NA	
B	14 (46.7%)	NA	
C	3 (10.0%)	NA	
Hemodynamics			
mRAP (mmHg)	4.74 ± 3.54	8.48 ± 5.49	<0.001
sPAP (mmHg)	80.46 ± 21.71	99.25 ± 24.81	<0.001
mPAP (mmHg)	46.54 ± 11.95	60.58 ± 15.03	<0.001
mPAWP (mmHg)	8.23 ± 3.59	8.44 ± 3.16	0.755
TPG (mmHg)	38.24 ± 11.21	52.38 ± 14.77	<0.001
Cardiac index (L/min/m^2^)	4.02 ± 1.59	2.47 ± 0.81	<0.001
PVR (wood unit)	7.08 ± 3.95	14.89 ± 7.11	<0.001
SVR (wood unit)	15.78 ± 7.37	22.01 ± 8.23	0.001
SvO_2_ (%)	65.50 ± 9.73	60.97 ± 10.00	0.004
Target therapy, *n* (%)			
ERAs	3 (10.1%)	29 (16.1%)	0.389
PDE-5is	19 (63.2%)	77 (42.7%)	0.036
Prostacyclin analogues	1 (3.3%)	14 (7.8%)	0.382
Combined therapies	3 (10.1%)	39 (21.7%)	0.139
No specific treatment	4 (13.3%)	21 (11.7%)	0.794
Laboratory variables			
Uric acid (*μ*mol/L)	366.57 ± 156.03	404.13 ± 125.95	0.149
BUN (mmol/L)	5.20 ± 1.81	5.51 ± 1.96	0.415
Creatinine (*μ*mol/L)	61.57 ± 21.00	67.23 ± 17.43	0.114
D-dimer (ng/ml)	399 (218, 1673)	149 (106, 202)	<0.001
International normalized ratio	1.31 ± 0.32	1.22 ± 0.43	0.315
NT-proBNP (pg/ml)	324 (81, 893)	739 (262, 1904)	0.004
BNP (pg/ml)	120 (80, 402)	221 (55, 427)	0.727
Arterial blood gas			
PH	7.45 ± 0.03	7.45 ± 0.03	0.522
PO_2_ (mmHg)	72.16 ± 13.72	72.13 ± 18.26	0.994
PCO_2_ (mmHg)	33.96 ± 11.68	28.43 ± 5.07	0.019
SO_2_ (%)	93.35 ± 5.13	93.00 ± 5.62	0.780
Blood cell counts (10^9^/L)			
WBC	4.18 ± 1.78	6.68 ± 2.09	<0.001
Neutrophils	2.26 ± 1.40	3.88 ± 1.79	<0.001
RBC	4.24 ± 0.85	4.92 ± 0.63	<0.001
PLT	114.73 ± 85.67	178.92 ± 72.54	<0.001
MELD scores	11 (8–13)	NA	

POPH, portopulmonary hypertension; IPAH, idiopathic pulmonary arterial hypertension; WHO FC, World Health Organization functional classification; PBC, primary biliary cirrhosis; sPAP, systolic pulmonary artery pressure; mRAP, mean right atrial pressure; mPAP, mean pulmonary artery pressure; mPAWP, mean pulmonary artery wedge pressure; PVR, pulmonary vascular resistance; TPG, transpulmonary gradient; SVR, systemic vascular resistance; SvO_2_, mixed venous oxygen saturation; ERAs, endothelin receptor antagonists; PDE-5is, phosphodiesterase 5 inhibitors; BUN, blood urea nitrogen; NT-proBNP, N-terminal pro brain natriuretic peptide; BNP, brain natriuretic peptide; PH, power of hydrogen; PO_2_, partial pressure of oxygen; PCO_2_, partial pressure of carbon dioxide; SO_2_, oxygen content of arterial blood; WBC, white blood cell; RBC, red blood cell; PLT, platelet; MELD score, model for end-stage liver disease scores; NA, not applicable. Hemodynamics were measured via right heart catheterization.

**Table 2:** Liver function test of portopulmonary hypertension and idiopathic pulmonary arterial hypertension at baseline.

Parameters	POPH (*n* = 30)	IPAH (*n* = 180)	*P* value
Total bilirubin (*μ*mol/L)	25.0 (17.0, 44.3)	19.0 (14.0, 28.3)	0.013
Direct bilirubin (*μ*mol/L)	10.5 (6.0, 17.0)	6.0 (4.0, 10.5)	<0.001
Albumin (g/L)	32.88 ± 4.27	38.41 ± 5.14	<0.001
ALT (IU/L)	26.5 (17.8, 38.0)	24.0 (19.0, 32.0)	0.566
AST (IU/L)	37.5 (30.0, 44.0)	25.0 (20.0, 30.0)	<0.001

POPH, portopulmonary hypertension; IPAH, idiopathic pulmonary arterial hypertension; ALT, alanine aminotransferase; AST, aspartate aminotransferase.

**Table 3:** Correlation between PVR and laboratory variables, liver function test, and therapies in POPH and IPAH.

	POPH		IPAH	
	*r*	*P*	*r*	*P*
Liver function test				
Total bilirubin	−0.394	0.031	0.218	0.003
Direct bilirubin	−0.364	0.048	0.178	0.018
AST	−0.136	0.474	0.139	0.066
D-dimer	0.009	0.964	0.016	0.913
Blood cell counts				
WBC	0.275	0.142	0.179	0.117
Neutrophils	0.394	0.031	0.107	0.167
NT-proBNP	0.203	0.299	0.438	<0.001
PCO_2_	0.014	0.940	0.004	0.976
Target therapies	−0.076	0.690	0.133	0.076
MELD scores (per unit)	−0.243	0.195	—	—

Data were conducted via Pearson or Spearman correlation analyses. PVR, total bilirubin, direct bilirubin, AST, D-dimer, blood cell counts, NT-proBNP, and PCO_2_ were regarded as continuous variables. Target therapies and MELD scores were deemed as categorical variables. PVR, pulmonary vascular resistance; POPH, portopulmonary hypertension; IPAH, idiopathic pulmonary arterial hypertension; AST, aspartate aminotransferase; WBC, white blood cell; NT-proBNP, N-terminal pro brain natriuretic peptide; PCO_2_, partial pressure of carbon dioxide; MELD scores, model for end-stage liver disease scores; —, not applicable.

**Table 4:** Independent determinants of PVR elevation from differential laboratory variables in POPH and IPAH.

	Independent predictors	*R* ^2^	Standardized *β*	95% confidence interval	*P* value
POPH	Neutrophils	0.156	0.394	0.109, 0.118	0.030
IPAH	NT-proBNP	0.187	0.433	0.001, 0.002	<0.001

Data were conducted via linear regression analyses. Models were adjusted by gases of blood (continuous variable), metabolic comorbidities (yes or not, categorical variable, including hypertension, diabetes, obesity, and dyslipidaemia), Child-Pugh score (categorical variable), and concomitant medications (categorical variable). PVR, total bilirubin, direct bilirubin, blood cell counts, and NT-proBNP were regarded as continuous variables. PVR, pulmonary vascular resistance; POPH, portopulmonary hypertension; IPAH, idiopathic pulmonary arterial hypertension; NT-proBNP, N-terminal pro brain natriuretic peptide.

## Data Availability

Data used to support the findings of this study are available from the corresponding author upon reasonable request.

## Additional Points

*Limitations*. First, the small sample size of POPH patients resulted from a single tertiary center with a referral difference. Patients who developed obvious cardiopulmonary symptoms and signs would come to our center for PH confirmation. However, most patients would turn to some general hospitals for treatment of primary liver disease. Second, because bilirubin is dynamically changing during disease development, data measured at baseline might not be the best indicators and long-term monitoring of bilirubin in POPH should be studied further.

## Supplementary Materials

Supplementary Materials We have collected related data from echocardiography because patients would like to accept a noninvasive test (echocardiography) rather than an invasive test (right heart catheterization) and found that there was no significant change between the baseline and last follow-up time in POPH. The escaped time between the baseline and the last follow-up time in POPH was 65.1 ± 17.3 months. Those data are summarized in Supplement Figure 1. Supplement Figure 1: Comparisons of indices measured by means of echocardiography between the time of baseline and the last follow-up in POPH, including sPAP (a), TAPSE (b), and LVEF (c). sPAP, systolic pulmonary artery pressure; TAPSE, tricuspid annular plane systolic excursion; LVEF, left ventricular ejection fractions.
