# Supplementary figures and images for: The Different Effects of Direct Bilirubin on Portopulmonary Hypertension and Idiopathic Pulmonary Arterial Hypertension
Source: Int J Clin Pract. 2022 Feb 3;2022:7021178. doi: 10.1155/2022/7021178 (PMC9159212; doi:10.1155/2022/7021178)

A

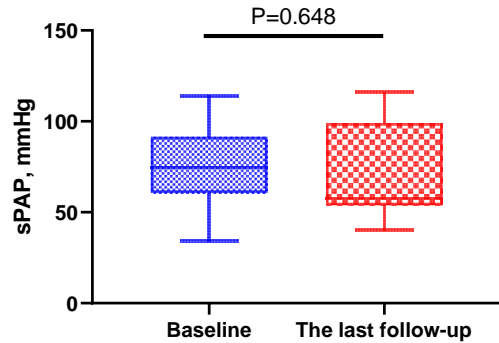

B

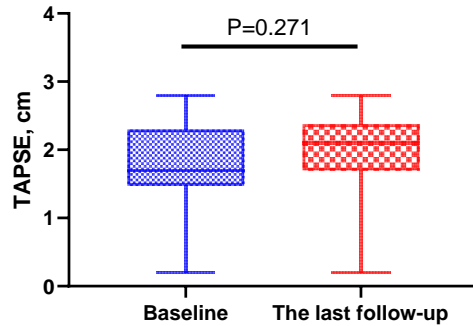

C

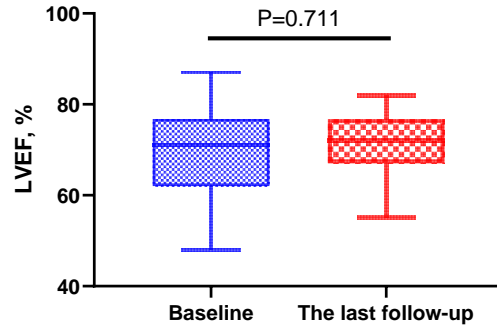

Supplement: Supplementary Materials — We have collected related data from echocardiography because patients would like to accept a noninvasive test (echocardiography) rather than an invasive test (right heart catheterization) and found that there was no significant change between the baseline and last follow-up time in POPH. The escaped time between the baseline and the last follow-up time in POPH was 65.1 ± 17.3 months. Those data are summarized in Supplement Figure 1. Supplement Figure 1: Comparisons of indices measured by means of echocardiography between the time of baseline and the last follow-up in POPH, including sPAP (a), TAPSE (b), and LVEF (c). sPAP, systolic pulmonary artery pressure; TAPSE, tricuspid annular plane systolic excursion; LVEF, left ventricular ejection fractions. [file 7021178.f1.pdf]
